# BA9 Transcriptomics in Huntington’s Disease 80-Gene Signature and MIR219A2-Linked Targets

**DOI:** 10.3390/ijms26188934

**Published:** 2025-09-13

**Authors:** Gözde Öztan, Halim İşsever, Levent Şahin

**Affiliations:** 1 Department of Medical Biology, Istanbul Faculty of Medicine, Istanbul University, Topkapı, 34093 Istanbul, Turkey; 2 Department of Public Health, Istanbul Faculty of Medicine, Istanbul University, Topkapı, 34093 Istanbul, Turkey; halimissever@gmail.com; 3 Department of Labor Economics and Industrial Relations, Faculty of Economics, Istanbul University, Fatih, 34126 Istanbul, Turkey; levent.sahin@istanbul.edu.tr

**Keywords:** Huntington’s disease, BA9, transcriptomics, miRNA–mRNA integration, *MIR219A2*, differential expression, Gene Ontology, KEGG, Reactome, STRING

## Abstract

Cortical transcriptional dysregulation is widespread in Huntington’s disease (HD). We re-examined prefrontal Brodmann Area 9 (BA9) RNA-seq (GSE64810; 20 HD, 49 controls) using BH-FDR and GEO2R to obtain differential-expression statistics for downstream in silico integration. A compact, direction-aware 80-gene panel was assembled for visualization/ranking only, while inference relied on validated target sets and full-universe testing. At FDR < 0.05, we detected Up = 2923 and Down = 2448 genes (ratio 1.19), indicating a mild predominance of up-regulation. MIR219A2 was strongly down-regulated, and four experimentally validated targets (FOXC1, NFKBIA, SLC38A2, SLC6A20) overlapped the up-regulated core; as expected for *n* = 4, no GO/KEGG/Reactome term met FDR < 0.05, and STRING returned no high-confidence edges. Beyond the curated panel, we tested MIR219A2 (hsa-miR-219a-5p; hsa-miR-219a-1-3p; hsa-miR-219a-2-3p) targets against the full FDR-significant BA9 up-regulated universe. Two orthogonal, experimentally supported resources—miRTarBase functional assays and ENCORI/starBase CLIP—showed direction-consistent, FDR-controlled enrichment, with effect sizes and uncertainty reported in the main text, supporting a BA9-specific, MIR219A2-aligned association signal. On the TF axis, MSigDB C3:TFT (gene symbols) revealed significant over-representation of TF target sets among BA9-Up under the same BA9 expressed-gene background after BH-FDR (e.g., NFAT motifs, C/EBP, FOXA/HNF3), while TRRUST v2 applied to the MIR219A2 CLIP–BA9-Up subset provided target-level transparency. MIR219A2 enrichments were robust to composition sensitivity analyses (marker-excluded and neuron/glia-stratified backgrounds). Exploratory GO–Biological Process bubbles are shown for trend summarization only; no term met FDR < 0.05 in the primary analysis. All conclusions are analysis-only; no wet-lab or biofluid/peripheral assays were performed, and findings are BA9-specific—generalization to other regions remains hypothesis-generating.

## 1. Introduction

### 1.1. Clinical and Genetic Background

Huntington’s disease (HD) is an autosomal-dominant neurodegenerative disorder characterized by an expanded CAG trinucleotide repeat in the HTT gene, resulting in a mutant polyglutamine huntingtin protein, which facilitates accurate molecular diagnosis and predictive counseling [1,2]. Genetic evidence further connects inherited repeat length and its somatic instability to age at onset and disease progression, establishing a quantitative basis for natural-history modeling and biomarker development [3,4,5]. HD usually appears in middle age, but it can happen at any age. The clinical heterogeneity of the disease is due to problems in distributed corticostriatal circuits rather than just a striatal lesion [6].

Repeat-category biology and inheritance dynamics influence clinical risk in addition to identifying the molecular lesion. Normal alleles (≤26 CAG) are stable, while intermediate alleles (27–35) are usually non-penetrant but unstable during meiosis. There is a known paternal bias toward expansion and clinical anticipation. Reduced-penetrance alleles (36–39) may or may not show up, while alleles with 40 or more CAGs are usually fully penetrant [7,8]. There is also a haplotype component to geographic prevalence differences: certain HTT haplogroups (such as A1/A2 in Europeans) are enriched among expansion chromosomes and probably influence local mutation rates, which helps to explain why East Asia has a significantly lower prevalence [9].

A significant advancement has been the acknowledgment that somatic CAG instability—not merely germline length—propels pathogenesis and alters the age of onset. The removal of the interrupting CAA(s) (“loss-of-interruption”) lengthens the uninterrupted CAG, accelerates somatic expansion, and is linked to an earlier onset, particularly in the reduced-penetrance range [10,11]. Large GWASs from the GeM-HD Consortium further identify DNA repair pathways (e.g., *FAN1*, *MSH3*, *PMS2*, *MLH1/3*, *LIG1*) as genetic modifiers of onset and progression, reinforcing a two-step model in which repeat expansion sets the stage for neuronal injury [12,13,14]. Recent work comparing somatic-expansion GWAS and clinical phenotypes underscores tissue-specific and cell-type-specific effects, with therapeutic implications for targeting mismatch-repair components [15,16].

From a practice standpoint, contemporary laboratory practice emphasizes accurate sizing and unambiguous reporting of the HTT CAG repeat, together with detection and documentation of interrupting sequence variants (e.g., CAA “loss-of-interruption”), to ensure correct classification and counseling [2,10,11]. Predictive testing therefore integrates repeat length, sequence context, family history, and counseling frameworks, acknowledging that polyglutamine length alone is an imperfect predictor at the individual level [17].

### 1.2. Cortical Involvement and the Brodmann Area 9 (BA9) Context

Structural MRI and task-based fMRI demonstrate early, topologically specific cortical thinning and altered prefrontal responses associated with executive dysfunction and inhibitory control, thereby connecting the dorsolateral prefrontal cortex (primarily BA9/46) to HD cognitive and neuropsychiatric phenotypes [18]. These cortical disturbances interact with striatal pathology via corticostriatal loops, a recognized pathway significantly involved in HD pathophysiology [19,20].

Longitudinal and cross-sectional MRI studies confirm progressive, regionally patterned cortical atrophy affecting dorsolateral prefrontal areas, correlating with clinical deterioration, thereby establishing BA9 involvement as prognostically significant rather than incidental [21]. Resting-state and task paradigms converge on dysconnectivity within executive control networks and diminished frontostriatal coupling across premanifest-to-manifest stages, establishing a functional link from BA9 physiology to cognition and behavior [22].

In vivo immune imaging provides additional evidence, as TSPO-PET shows heightened cortical microglial activation in premanifest gene carriers, with increases that correlate with peripheral cytokines and indicate a widespread inflammatory environment early in the disease progression [23].

Electrophysiological readouts align with disrupted frontal microcircuits, while transcranial magnetic stimulation (TMS) studies indicate abnormal excitability and diminished inhibitory tone, which are anticipated to compromise executive control [24,25]. Recent human data at the cellular level demonstrate the selective vulnerability of layer-5a corticostriatal projection neurons, establishing a mechanistic connection between BA9 pyramidal pathology and subsequent striatal disconnection [26]. Finally, studies of structural connectomics show that long, expensive white matter connections are especially weak in HD. This phenomenon is because the corticostriatal pathways get weaker over time, which matches the risk of macro-scale network problems with BA9-centered circuit problems [27,28,29].

### 1.3. Cortical Transcriptomics: Broad Themes from BA9

Bulk mRNA-seq of human BA9 (GSE64810; 20 HD, 49 controls) reported a mild predominance of up-regulation, together with enrichment of immune/neuroinflammatory and developmental (homeobox/HOX) programs; marker patterns across multiple brain cell types indicated a broad tissue-level response. These characteristics have established GSE64810 as a benchmark dataset for cortical re-analyses [30]. Subsequent epigenomic and transcriptomic investigations substantiated developmental and immune pathways in the HD cortex, highlighting modified H3K4me3 landscapes in prefrontal neurons [31].

Bulk BA9 transcriptomics consistently exhibits a predominant up-regulation signal characterized by significant immune/inflammatory representation and transport/metabolic components. This pattern is replicated in independent meta-analyses of human HD brain datasets and cross-tissue comparisons that delineate robust brain subnetworks in HD [32]. In addition to immunity, BA9 datasets consistently reveal developmental programs, including HOX-associated axes. In the prefrontal cortex, members of the HOX-cluster miRNA/mRNA neighborhood (e.g., the miR-10 family alongside adjacent HOX genes) are co-upregulated, indicating ectopic activation of developmental modules within the adult cortex. This is in line with lifespan single-cell atlases of the human prefrontal cortex that show tightly controlled developmental paths that could leave strong transcriptional marks in adulthood if they are reactivated or released from repression [33,34].

The convergence with epigenomic data substantiates a chromatin-level foundation for these cortical signatures. Neuron-enriched BA9 chromatin exhibits extensive modifications of promoter-associated H3K4me3 and indications of Polycomb/PRC2 state transitions, aligning with gene-regulatory transformations that can simultaneously enhance immune and developmental responses; comprehensive analyses contextualize these results within a unified HD epigenetic framework [31,35].

While bulk profiles provide an average across cell types, single-nucleus and multi-omic studies demonstrate that astrocytes and microglia significantly influence the cortical signal, exhibiting regional variability depending on state. This supports the notion that the up regulation of BA9 partially signifies glial activation in conjunction with neuronal stress.

Astrocytic phenotypes, linked to the severity of disease, have become clearer through recent data from multiple regions of the human brain. This supports the idea that glial cells play a role in cortical dysregulation [36,37]. At the transcript level, bulk cortical RNA-seq in HD shows isoform-level remodeling, with widespread alternative splicing in the human cortex that adds to gene-level findings and shows that BA9 has more post-transcriptional control [38].

### 1.4. Multicellular Remodeling in Human HD Cortex

Single-nucleus RNA-seq reveals disease-associated states in astrocytes, oligodendrocytes, microglia, and neurons, suggesting that bulk profiles indicate multicellular remodeling rather than a singular neuronal process [36]. Complementary studies emphasize astrocyte molecular signatures in HD and their possible role in circuit dysfunction [39]. Parallel mechanistic studies indicate that neuroimmune pathways, particularly microglia-complement-mediated synapse loss, serve as initial factors in corticostriatal circuit pathology and cognitive dysfunction [40].

In addition to these observations, single-nucleus and cross-species studies demonstrate halted maturation in the oligodendrocyte lineage throughout the human cortex, characterized by transcriptionally immature OL/OPC states that are consistent across regions and can affect myelination phenotypes [41,42]. Independent glia-centric re-analyses converge on astrocytic and oligodendroglial remodeling, encompassing stress-response programs and chaperone up-regulation that are consistently observed across HD datasets, thereby reinforcing a coordinated, non-neuronal component to cortical dysregulation [43]. Multi-omic integration elucidates disparate astrocyte states associated with inflammatory and metabolic axes, a categorization that likely corresponds to unique circuit effects in the prefrontal cortex [44].

Recent human sorting-based and snRNA-seq profiling reveals that layer 5a corticostriatal projection neurons exhibit selective vulnerability, thereby establishing a specific pyramidal subpopulation associated with early corticostriatal disconnection and creating a mechanistic link between cortical pathology and striatal dysfunction [26]. Emerging human and model evidence suggests that endothelial dysfunction and neurovascular-unit imbalance, including abnormalities related to the blood–brain barrier (BBB), represent additional layers of cortical remodeling that interact with astrocytic and microglial programs in HD [45,46].

Collectively, these findings endorse a multicellular model of BA9 pathology characterized by neuron-intrinsic vulnerability alongside glial (astrocyte/oligodendrocyte) reprogramming and vascular involvement, thereby enhancing bulk BA9 profiles and necessitating cell-resolved and spatial follow-up in the human cortex [41,43].

### 1.5. MicroRNAs in HD and the Biological Rationale for MIR219A2

MicroRNAs (miRNAs) are powerful post-transcriptional regulators increasingly associated with neurodegeneration and HD brain signatures. The miR-219 family is essential for oligodendrocyte differentiation and myelination; the absence of miR-219 disrupts maturation in vivo, situating this axis at the convergence of glial biology and cortical function [47,48]. Considering the mounting evidence of glial involvement in the HD cortex, focusing on a miR-219-centered mechanism provides a biologically substantiated pathway to link miRNA alterations with directionally consistent mRNA responses in BA9 [36].

In addition to its role in development, miR-219 is abundant in human oligodendrocytes and continues to be expressed in the adult cortex, facilitating both myelin maintenance and maturation [49]. In demyelinating conditions, miR-219 is consistently diminished in human lesions, and experimental enhancement of miR-219 facilitates remyelination and enhances cognitive and functional outcomes in vivo, highlighting its reparative capacity [50,51]. Mechanistically, miR-219 contributes to a myelin miRNA axis with miR-338; genetic and functional data demonstrate that these miRNAs work together to promote the development of oligodendrocyte lineages and myelin formation, offering a tenable mechanism by which minor variations in miR-219 dosage may influence the physiology of the cortical circuit [52].

At the target level, miR-219 has been associated with the regulation of oligodendrocyte precursor cell (OPC) mitogenic and anti-myelinating pathways (e.g., *PDGFRA*, *SOX6*), providing a definitive pathway from miRNA dysregulation to oligodendroglial state transitions and modified neuron–glia interactions in the prefrontal cortex [53]. At the same time, HD brains show widespread miRNA disturbance, with changes in editing and abundance in many miRNA families. This means that any changes to *MIR219A2* are part of a larger post-transcriptional landscape that is important to BA9 biology [54]. *MIR219A2* is a biologically relevant candidate for integrative analysis in the HD cortex: it is linked to oligodendrocytes, modulates programs essential for myelination and glia-neuron support, and exists within a transcriptomic framework where miRNA-directed regulation is already disrupted [50,52,54].

### 1.6. Analytical Strategy: BA9 Re-Analysis and a Compact, Direction-Aware Core Signature

The public availability of raw/processed BA9 data facilitates reproducible pipelines and transparent re-analysis [31]. To achieve a balance between interpretability and statistical power, we organize downstream work around a compact, direction-aware core (FDR-significant genes that maintain up/down), instead of an unfiltered omnibus list.

This enables coherent enrichment and network analyses and facilitates miRNA–mRNA integration where directionality holds biological significance (i.e., reduced miRNA expression is anticipated to relieve repression and subsequently elevate the abundance of its direct mRNA targets). Enrichment resources (GO/KEGG/Reactome) and interaction evidence (STRING) offer complementary context for this core [55].

### 1.7. Positioning Within Current HD Biology

The BA9 immune/developmental signature correlates with extensive literature on neuroinflammation, NF-κB-associated glia-neuron interactions, and complement-driven synaptic remodeling in HD models and human tissue [56,57]. Recent multi-omic and single-cell studies underscore astrocytic and oligodendroglial states that may intersect with miR-219 biology and metabolic/vascular support functions in the cortex [37]. The field is moving toward biological staging and region-resolved mechanisms, which shows how important it is to have standardized cortical signatures that can be combined with validated miRNA targets [3].

Even though there are strong cortical transcriptomic themes, there are still some big questions: (i) which miRNA changes in human BA9 are linked to validated targets that change in a way that is consistent with the direction; (ii) whether compact, direction-aware gene sets still have enough power to show specific pathways and network modules without becoming too general; and (iii) how these overlaps fit into cellular programs (like astroglial, oligodendroglial, and neurovascular) that single-cell data suggests are involved [36,58].

We re-analyze BA9 (GSE64810) with strict FDR control, establish an 80-gene, direction-aware core signature, and assess whether miRNA dysregulation—focusing on *MIR219A2* as a biologically relevant candidate—correlates with directionally consistent alterations in experimentally validated targets (DIANA-TarBase v9.0 2025). We subsequently contextualize overlaps with FDR-controlled enrichment (GO/KEGG/Reactome) and high-confidence PPI (STRING) to provide a clear, mechanism-based representation of HD BA9 appropriate for figure-level reporting and hypothesis formulation [58,59,60].

To address concerns about novelty in re-analyses of GSE64810, we explicitly adopt an unbiased, full-universe integration: validated miRNA targets are tested against the complete FDR-significant BA9 changes, and statistical claims are reported with effect sizes and uncertainty (OR, 95% CI, FDR). This design minimizes panel selection bias and elevates the analysis beyond descriptive overlap.

Given known regional heterogeneity in HD, we deliberately focus on the prefrontal cortex (BA9) to minimize confounding by severe neurodegeneration, and we do not extrapolate our conclusions to the striatum or motor cortex. The analytical workflow is general and can be applied to additional regions in future work.

This work presents an analysis-only re-examination of BA9 RNA-seq data. Accordingly, we make no causal or clinical inferences; rather, we aim for computational convergence (agreement across methods and thresholds) as the immediate objective. Such convergence is intended to guide and de-risk subsequent experimental follow-up and biofluid/peripheral validation.

## 2. Results

### 2.1. Global Differential Expression in BA9 (GSE64810)

**Figure 1:**
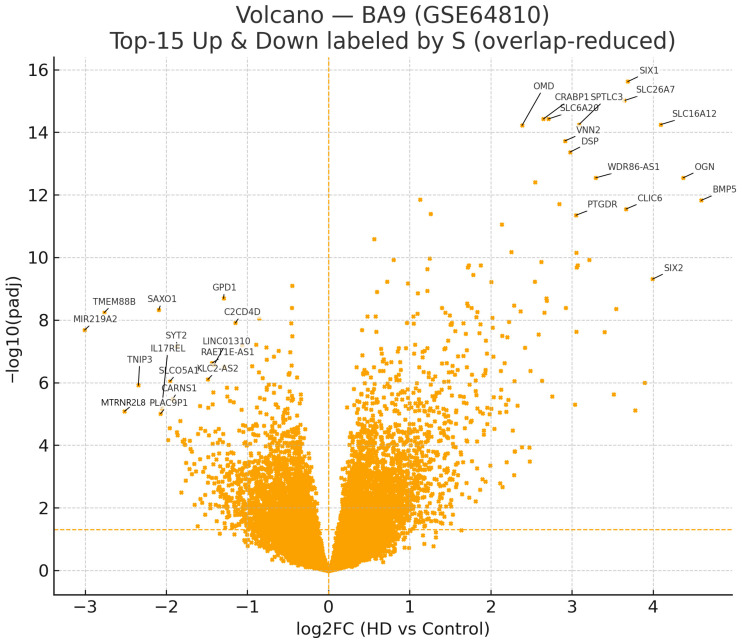
BA9 (GSE64810) volcano plot of differential expression. FDR < 0.05 yields Up = 2923, Down = 2448 (ratio 1.194). The horizontal dashed line marks padj = 0.05; the vertical dashed line indicates log2FC = 0. To aid interpretation without clutter, top-15 up and top-15 down genes by S = |log2FC| × (−log10(padj)) are labeled with minimized overlap. Global counts are descriptive; inference relies on pathway/regulatory analyses. Top 15 labels by S (heuristic; display only).

We analyzed the GSE64810 mRNA-seq dataset generated from post-mortem BA9 (prefrontal cortex), comprising 20 HD and 49 control samples. Following the series-level normalization and our predefined significance criterion (padj < 0.05), we reassessed the transcriptome-wide changes to establish a baseline HD signature for downstream analyses. Differential-expression statistics were computed with GEO2R (NCBI GEO) using series-matrix inputs and Benjamini–Hochberg FDR control; the volcano plot (Figure 1) and Top-15 labels were generated directly from the GEO2R-exported results table.

At FDR < 0.05, we detected Up = 2923 and Down = 2448 genes (ratio 1.194), indicating a mild excess of up-regulated genes. A volcano plot displays significant up-regulated genes in red and significant down-regulated genes in blue, with non-significant genes in light gray. To anchor the signal, we annotated the top 15 up- and top 15 down-regulated genes by a composite score (|log2FC| × −log10(padj)). Consistent with prior observations, several highly significant up-regulated transcripts occupy the upper-right quadrant, whereas fewer down-regulated genes reach comparable significance in BA9.

All analyses reported here refer to BA9; we do not infer cross-regional generalizability from these results. To place the BA9 signal in a cellular context, we annotated the FDR-significant sets with canonical CNS marker panels (astrocyte, microglia, oligodendrocyte, endothelial, and neuron). This analysis is descriptive and intended to indicate potential composition-linked programs rather than cell-intrinsic regulation (Supplementary Table S8). These annotations are descriptive and intended to provide composition context rather than cell-intrinsic regulation; definitive cellular attribution will require single-nucleus or spatial transcriptomics.

### 2.2. Top-Ranked Differentially Expressed Genes

To contextualize Figure 1 with gene-level detail, the top 15 up- and top 15 down-regulated lists—derived from the GEO2R results and ranked by the composite score S (Methods)—are provided in Table 1 and Table 2. These tables report Symbol, log2FC, padj (BH), and S for each gene.

We chose a core set of 80 genes at FDR < 0.05 ranked by the composite score for downstream miRNA–mRNA integration that focused on a single miRNA. Of these, 74 were up-regulated and 6 were down-regulated, which is what we would expect from the known immune/inflammatory bias in BA9 that is linked to HD (see Supplementary Tables S1 and S2). This set strikes a balance between statistical power for enrichment/network analysis and readability in figures. It also allows for directionally coherent testing, such as when miRNA up-regulation predicts down-regulation of its direct targets.

### 2.3. The 80-Gene Core Signature

Applying the S-based priority rule yielded an 80-gene core set comprising 74 up-regulated and 6 down-regulated transcripts. Directionality was preserved so the set can serve as a backbone for direction-aware downstream analyses. The complete core list, with up/down partitions, is provided in Supplementary Tables S1 and S2. Its composition mirrors the BA9 landscape previously highlighted for GSE64810—namely immune/neuroinflammatory and developmental/HOX programs—supporting a broad, multicellular response in HD BA9.

### 2.4. MIR219A2 Down-Regulation in BA9 and Target Overlaps with BA9 Up/Down Sets

*MIR219A2* was markedly down-regulated in BA9 (GEO2R statistics: log2FC = −3.005; padj = 2.08 × 10^−8^), consistent with de-repression of its direct mRNA targets. We overlapped experimentally validated targets of hsa-miR-219a-5p, hsa-miR-219a-1-3p, and hsa-miR-219a-2-3p (DIANA-TarBase exports) with the up-regulated component of the 80-gene BA9 signature, yielding four targets—*FOXC1*, *NFKBIA*, *SLC38A2*, and *SLC6A20*—each up-regulated in BA9 (Table 3; the full up-regulated partition of the 80-gene core appears in Supplementary Table S1). No validated MIR219A2 targets overlapped with the down-regulated subset of the 80-gene core (*n* = 0). The complete down-regulated partition of the core signature is provided in Supplementary Table S2.

Given the overlap size (*n* = 4), inferential power is inherently limited; accordingly, we do not claim pathway enrichment or network structure at FDR < 0.05. We therefore present directionality at the gene level and treat ORA/PPI outputs as exploratory.

### 2.5. Enrichment and PPI Analysis of the Four-Gene Overlap

We intersected experimentally validated MIR219A2 targets (mature forms: hsa-miR-219a-5p, hsa-miR-219a-1-3p, and hsa-miR-219a-2-3p from DIANA-TarBase exports) with the up-regulated component of the BA9 80-gene core. The overlap comprised four genes—*FOXC1*, *NFKBIA*, *SLC38A2*, and *SLC6A20* (Table 3)—while no validated targets overlapped the down-regulated subset (*n* = 0).

To assess pathway context, we performed over-representation analysis (ORA) against GO, KEGG, and Reactome with Benjamini–Hochberg FDR control, using the tested BA9 gene universe as background, a term-size range of 5–500 genes, and a minimum overlap of 2. In parallel, we queried STRING (Homo sapiens, combined evidence channels) with a minimum required interaction score of 0.7 (high confidence) and inspected putative modules and hubs.

**Figure 2:**
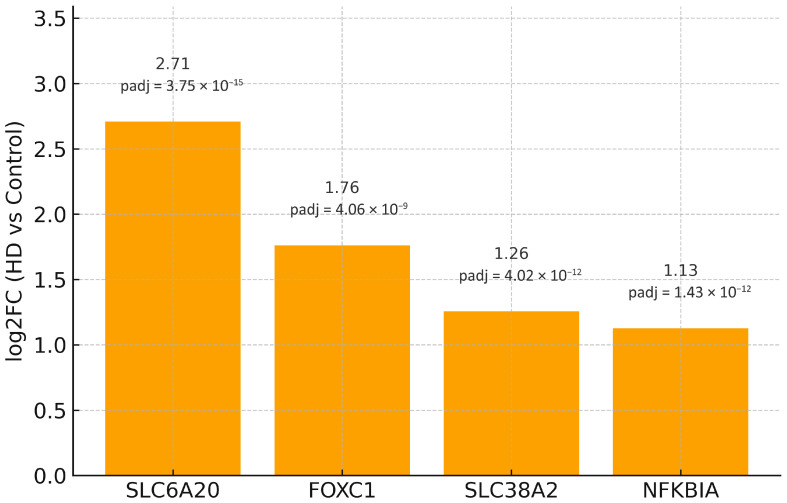
Log2FC (HD vs. Control) for the four validated *MIR219A2* targets overlapping the BA9 up-regulated core (*FOXC1*, *NFKBIA*, *SLC38A2*, *SLC6A20*). Per-bar labels indicate log2FC (top) and adjusted *p* values (bottom). Source data for Figure 2 are provided in Supplementary Table S7. The data is presented for the purpose of directionality and should not be used for inferential claims.

As expected for *n* = 4, statistical power was limited. In the primary analysis, no GO/KEGG/Reactome term passed FDR < 0.05, and STRING returned no high-confidence edges among the four nodes at score ≥ 0.7; accordingly, a cohesive subnetwork or hub architecture could not be established at this stringency. For transparency, Figure 2 shows a bar plot of log2FC for the four overlapping targets, each up-regulated in BA9, directionally consistent with MIR219A2 down-regulation.

Formal enrichment tests applied to the 80-gene core did not reach significance at FDR < 0.05, consistent with the limited power of an overlap of *n* = 4. Because these four overlapping genes are known to be multi-regulated (e.g., by transcription factors such as SMAD4, NFATC1, KDM5B, and BACH1), the overlap should be interpreted as consistent with—rather than uniquely diagnostic of—MIR219A2-mediated de-repression in BA9. We therefore refrain from assigning causality to MIR219A2 in bulk tissue.

**Figure 3:**
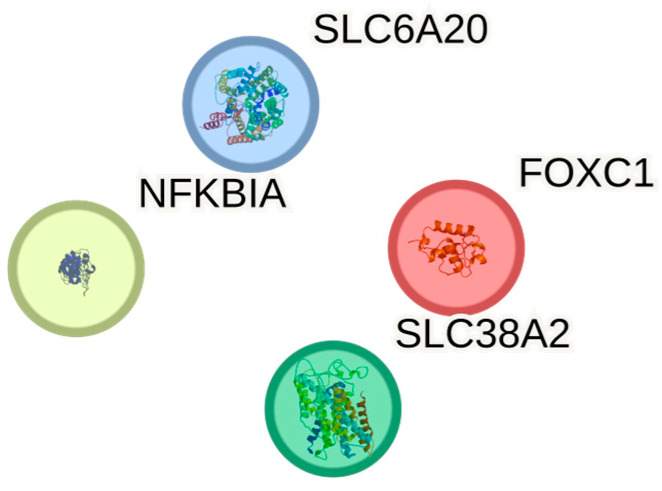
Node-only layout for the four overlapping *MIR219A2* targets. No high-confidence STRING edges (score ≥ 0.7) were returned; accordingly, no interactors were added, and nodes are shown disconnected for transparency.

Figure 3 depicts the four validated *MIR219A2* targets that overlap the BA9 up-regulated set (*FOXC1*, *NFKBIA*, *SLC38A2*, *SLC6A20*) as isolated nodes because no high-confidence STRING edges (minimum required interaction score ≥ 0.7) were returned among them under the prespecified settings (Homo sapiens, combined evidence channels). Disconnected nodes are displayed for completeness, with HUGO Gene Nomenclature Committee (HGNC) symbols as labels; no first-shell interactors were added, and no edges are drawn. The absence of edges at this stringent threshold indicates that these proteins do not form a cohesive physical interaction module supported by high-confidence evidence, which is consistent with their diverse molecular roles (a transcription factor, an NF-κB pathway inhibitor, and two solute transporters). Importantly, a lack of direct PPI edges does not preclude indirect relationships (e.g., shared regulators or pathway crosstalk) and is expected for small overlaps (*n* = 4).

**Figure 4:**
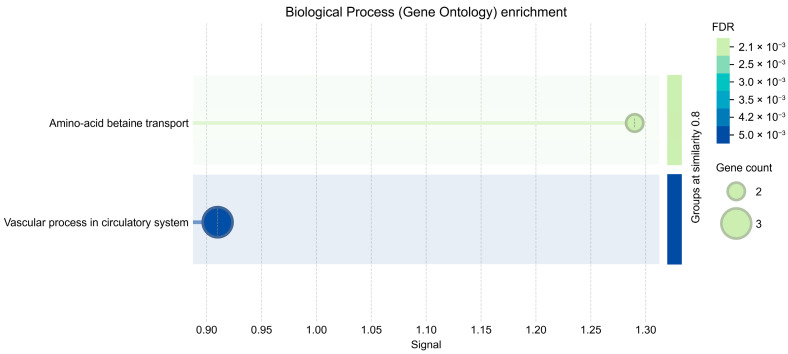
Exploratory STRING GO–Biological Process visualization for the four validated *MIR219A2* targets that overlap BA9 up-regulated genes (GSE64810). Bubble size denotes the number of overlapping genes; color denotes STRING q-values. Terms were redundancy-reduced by semantic similarity (threshold 0.8), and relaxed display thresholds were used for visualization only (default STRING background). Primary inferential ORA used the BA9 expressed-gene background with one-sided Fisher and Benjamini–Hochberg FDR across GO/KEGG/Reactome; no GO-BP term met FDR < 0.05 in that analysis (see Supplementary Table S10). Given the small overlap (*n* = 4), these signals are exploratory and hypothesis-generating.

In an exploratory GO–Biological Process summary that applies semantic similarity reduction to deduplicate terms, a small number of BP categories reached FDR < 0.01 but involved only 2–3 genes per term and should therefore be interpreted cautiously (Figure 4). For clarity, the exploratory GO-BP bubbles (Figure 4) were generated using relaxed parameters for visualization; no term passed FDR < 0.05 in the primary analysis, and those signals should be considered hypothesis-generating. Bubble plots were produced under relaxed display thresholds in STRING to summarize trends; inferential calls relied on our primary pipeline (expressed-gene background, BH-FDR), where no GO-BP term was significant at FDR < 0.05.

For transparency, the parameters and outcome of the primary-pipeline GO-BP ORA are summarized in Supplementary Table S10 (no term met FDR < 0.05). Although the overlap is biologically coherent—spanning a transcription factor (*FOXC1*), an NF-κB pathway regulator (*NFKBIA*), and solute transporters (*SLC38A2*, *SLC6A20*)—the set size precludes statistically robust enrichment or network structure under stringent thresholds. Applying the same workflow to the full padj < 0.05 up-regulated universe (beyond the 80-gene core) is expected to increase overlap counts, reveal FDR-significant GO/KEGG/Reactome terms, and uncover STRING modules and hubs suitable for figure-grade network panels.

ORA background was set to all tested BA9 genes; multiple testing was controlled by BH FDR; term-size bounds were 5–500 genes per term; the minimum overlap for testing was two genes. STRING was queried for Homo sapiens with combined evidence channels at a high-confidence threshold of 0.7; network statistics inspected included degree and betweenness, although no hubs were reported due to the absence of edges.

### 2.6. Full-Universe MIR219A2 Target Enrichment (Panel-İndependent)

We tested *MIR219A2* (hsa-miR-219a-5p) targets against the full FDR-significant up-regulated BA9 universe (background = expressed genes). Two independent resources were used: miRTarBase functional-only (luciferase/qPCR/Western; *n* = 8) and ENCORI/starBase CLIP-supported (AGO-CLIP ≥ 1; *n* = 640), alongside a validated-any union (*n* = 40). Enrichment was assessed by one-sided Fisher’s exact test with Benjamini–Hochberg FDR across the three pre-specified tests.

This full-universe test directly addresses the limited power of the four-gene overlap by evaluating validated *MIR219A2* targets against all FDR-significant up-regulated BA9 genes; effect sizes, 95% CIs, and FDR-adjusted *p* values are reported (Table 4).

Although no wet-lab validation was undertaken in this re-analysis, the *MIR219A2* signal satisfies our computational validation criteria: (i) replication across two orthogonal resources (functional assays curated in miRTarBase and AGO-CLIP interactions in ENCORI/starBase), (ii) direction-aware consistency (down-miRNA vs. up-mRNA), and (iii) robustness to composition confounding (marker-excluded and stratified backgrounds; Supplementary Table S9). Clinical translation (biofluids/peripheral tissues) was not undertaken here and is deferred to future work; concrete predictions and assay plans are detailed in the Discussion (Section 3).

As a sensitivity check for composition confounding, we re-tested enrichment after excluding the union of canonical CNS markers and within neuron- versus glia-enriched backgrounds; direction-aware signals persisted (Supplementary Table S9).

For transparency and reproducibility, the CLIP-supported analysis is provided in Supplementary Table S11a (MIR219A2 CLIP–BA9 Up overlap). The table reports the 2 × 2 contingency counts (a, b, c, d) computed on the BA9 expressed-gene background, the full list of overlapping genes with their BA9 statistics (gene symbol, log2FC, padj), and effect-size estimates with uncertainty (odds ratio with Haldane–Anscombe correction, 95% CI), together with the one-sided Fisher p and BH-FDR q values. Set sizes (n) and observed overlaps (a) for the MIR219A2 CLIP target set are also listed. All calculations mirror the primary inferential pipeline described in 2.6/Methods (one-sided Fisher, BA9 expressed-gene universe, FDR control across the three pre-specified tests). These outcomes indicate a statistically grounded, panel-independent *MIR219A2* signal in BA9. See Tables S3–S6 for overlapping genes and summary statistics. Because the analysis was performed in BA9, enrichment calls are region-specific and should not be assumed to generalize to the striatum or motor cortex.

As a sensitivity check for composition confounding, we re-tested *MIR219A2* target enrichment after excluding the union of cell-type marker genes from the background and, separately, within neuron-enriched and glia-enriched subsets. The direction-aware enrichment remained qualitatively similar, indicating that the signal is not solely driven by canonical marker genes (Supplementary Table S9).

### 2.7. TF Co-Regulation Signals (MSigDB C3:TFT; BA9 Expressed-Gene Background)

Within the MIR219A2 CLIP-overlap subset (*n* = 138), TRRUST listed key regulators with multiple targets (Supplementary Table S12); these are treated as supportive and exploratory. Using the BA9 expressed-gene background for formal inference, MSigDB C3:TFT showed significant TF-target over-representation among BA9 Up after FDR control (e.g., UBN1_TARGET_GENES: OR = 1.79, 95% CI [1.57, 2.03], q = 8.34 × 10^−15^; HES2_TARGET_GENES: OR = 1.85, 95% CI [1.60, 2.15], q = 3.90 × 10^−12^; TGGAAA_NFAT_Q4_01 [NFAT motif]: OR = 1.67, 95% CI [1.47, 1.89], q = 8.45 × 10^−12^; NFAT_Q6: OR = 2.92, 95% CI [2.18, 3.90], q = 4.16 × 10^−9^; CEBPB_02: OR = 2.68, 95% CI [2.02, 3.54], q = 2.57 × 10^−8^; TGTTTGY_HNF3_Q6 [FOXA/HNF3 motif]: OR = 1.91, 95% CI [1.58, 2.30], q = 3.07 × 10^−8^). Full results are provided in Supplementary Table S11b.

## 3. Discussion

Our re-analysis of BA9 shows that the main transcriptional structure of HD is unchanged, with a strong FDR-controlled tilt toward up-regulation and enrichment of immune/neuroinflammatory and developmental (HOX/homeobox) programs. This closely mirrors the original GSE64810 report and other studies of cortical transcriptomics [58]. This global pattern calls for concise, direction-aware summaries—such as an 80-gene core that preserves up- and down-directionality—so that enrichment and network follow-ups can be interpreted coherently. Within the down-regulated subset, *MIR219A2* shows a substantial effect size and high significance, consistently correlating with up-regulation of several experimentally validated mRNA targets in BA9. miR-219 is critical for oligodendrocyte differentiation and myelination, suggesting that its reduction may hinder maturation and myelin maintenance in the cortex—a process increasingly linked to HD susceptibility [47,48]. Cell-type-resolved research further demonstrates that astroglial and oligodendroglial programs are modified in the HD cortex, aligning with a multicellular response that underpins bulk BA9 signals [36,43].

We emphasize that bulk BA9 profiles conflate cell-type composition and cell-intrinsic regulation. Our marker-aware context and sensitivity checks mitigate—but do not eliminate—this limitation; accordingly, the inferred *MIR219A2* signal should be considered hypothesis-generating with respect to cellular provenance. Definitive attribution will require single-nucleus or spatial transcriptomics and targeted functional assays. Alternative regulatory explanations remain plausible; distinguishing miRNA-driven effects from TF-mediated or epigenetic regulation will require perturbational, cell-resolved assays.

The validated *MIR219A2* overlap within the 80-gene core (*n* = 4) is biologically coherent yet underpowered for FDR-significant pathway or network calls at stringent thresholds. To address this, we complemented the core-based view with a powered, panel-independent analysis against the full FDR-significant BA9 up-regulated universe (Section 2.6; Table 4), which yields direction-consistent, statistically significant enrichment with explicit effect sizes and uncertainty.

We note that enrichment estimates based on large predicted-target universes (e.g., DIANA/MicroT) likewise indicate limited power for *n* = 4 on the curated core. Our primary inference, therefore, relies on validated target resources (miRTarBase, ENCORI) and an expressed-gene background, gaining power in the full-universe test while preserving specificity (Section 2.6; Table 4).

To mitigate selection bias and maximize interpretability, we analyzed validated miRNA–mRNA relationships against the full set of FDR-significant transcriptional changes in BA9. Two independent evidence streams—functional assays curated in miRTarBase and AGO-CLIP interactions from ENCORI/starBase—were evaluated separately, and results are reported with effect sizes and uncertainty (odds ratios, 95% confidence intervals, FDR-adjusted *p*-values). The concordant, direction-aware enrichment observed across these orthogonal resources supports a *MIR219A2*-centered regulatory signal in BA9; nevertheless, the findings should be regarded as hypothesis-generating pending experimental confirmation.

Four genes—*FOXC1*, *NFKBIA*, *SLC38A2*, and *SLC6A20*—were increased in HD BA9 when validated *MIR219A2* targets from DIANA-TarBase were intersected with the up-regulated arm of the 80-gene BA9 core. Each points to a mechanistic axis of interest. NFKBIA encodes IκBα, a negative regulator of NF-κB; its increase may reflect compensatory feedback to chronic inflammatory activation, a putative driver of HD pathogenesis [61,62]. *FOXC1* participates in pericyte/endothelial programs and has been associated with cerebrovascular development and barrier-related phenotypes, corresponding with emerging neurovascular contributions to HD [63]. *SLC38A2* (*SNAT2*) is dynamically regulated by nutrient/stress cues and localizes to neurons and perivascular astrocytic end-feet, indicating altered amino-acid transport/metabolic signaling in BA9 [64,65]. Glycine/proline transport and NMDAR co-agonism modulation by *SLC6A20* link solute transport to synaptic physiology, a recurring theme in HD circuit dysfunction [66].

Methodologically, high-stringency network and pathway tests behaved as expected for *n* = 4. Over-representation analyses (GO/KEGG/Reactome; BH-FDR; background = tested BA9 genes; term size 5–500; minimum overlap = 2) returned no FDR-significant categories, and STRING (Homo sapiens; combined evidence; score ≥ 0.7) reported no edges among the four nodes; we therefore documented directionality and provided a node-only schematic as transparent, hypothesis-generating visuals. While small, the overlap is directionally coherent—miRNA down, targets up—and mechanistically anchored across inflammatory, neurovascular, and synaptic/metabolic facets.

These BA9 results fit well within the larger HD literature. Complement-tagged synapse loss and cortical immune activation have been demonstrated in human tissue and models, situating our immune-skewed BA9 profile in a broader neuroimmune context. Blood–brain concordance studies and multi-omic/single-cell research underscore immune and glial programs (including protective astrocyte states), thereby reinforcing a systems-level perspective rather than a neuron-centric lesion [30]. Functional enrichment emphasizes developmental/homeobox (HOX) programs alongside immune and neuroinflammatory signals, indicating that dysregulated developmental pathways endure in the adult cortex and interact with immune and glial processes [58].

The overlap indicates a neurovascular–metabolic–synaptic interface in BA9. A neuro-vascular component is plausible given growing evidence of endothelial/pericyte involvement and blood–brain barrier (BBB) dysfunction in HD, positioning vascular biology alongside established neuronal mechanisms [45]. Within the broader neurovascular unit (NVU), coordinated signaling among endothelium, pericytes, astrocyte end-feet, and neurons is essential for cortical function, offering a framework in which BA9 transcriptional shifts may reflect coupled vascular–glial–neuronal adaptation [67]. Additionally, transporter signals point to changed amino acid handling at the NVU.

According to astrocyte–neuron shuttling, which can adjust local metabolism and neurotransmission in the cortex, neutral amino acid carriers of the SLC38 family, including *SNAT2* (*SLC38A2*), are found at barrier and parenchymal interfaces [68]. A specific mechanism by which transporter perturbation could affect prefrontal network excitability pertinent to executive function is provided by *SLC6A20*, which controls brain glycine homeostasis at the synapse and thereby modulates NMDAR co-agonism [66].

The pattern is consistent with an immune tone that is continuously activated yet buffered by negative feedback from a regulatory standpoint. Changes in the components of the NF-κB pathway likely intersect with the immune-skewed cortical landscape because microglial NF-κB signaling is an inducible driver of neuroinflammation and shapes state transitions that propagate cytokine/chemokine programs [69]. The interpretation will depend on the cellular context. Combining benchmarking-guided method selection to reduce biases in reference construction with bulk-to-single reference deconvolution (e.g., CIBERSORTx) to estimate cell-type contributions and expression shifts is a feasible approach [70,71]. Spatially resolved transcriptomics and high-plex RNA imaging can subsequently assess whether directionally coherent alterations co-localize within the NVU or specific cortical layers in Brodmann area 9, thereby reinforcing causal inferences regarding cellular neighborhood [40]. Regarding cellular provenance, the present bulk BA9 analyses cannot ascribe regulation to specific cell types. Our marker-based sensitivity checks (Supplementary Table S9) suggest that the *MIR219A2* signal is not solely explained by gross composition shifts; however, single-nucleus or spatial assays will be required to localize dysregulation to glial, vascular, or neuronal compartments.

Mechanistic predictions are testable in human systems. In iPSC-derived glial and mixed neuron–glia models, targeted manipulation of transporter activity or pathway regulators is expected to alter NMDA-related physiological and metabolic readouts in alignment with BA9 signatures; concurrent AGO-CLIP or reporter assays can directly validate priority miRNA–target pairs [66]. Translationally, vascular and transporter-linked markers in CSF/plasma, along with imaging metrics sensitive to perfusion and BBB integrity, provide orthogonal modalities to triangulate the cortical signal and assess its generalizability across regions and cohorts [72,73].

Although no biofluid/peripheral analyses were performed in this re-analysis, the present results specify falsifiable predictions and an assay plan: (i) miRNA—*MIR219A2* should be decreased in CSF, plasma, and/or PBMCs; quantify by small-RNA-seq or RT-qPCR with spike-in and hemolysis controls; (ii) mRNA—*FOXC1*, *NFKBIA*, *SLC38A2*, and *SLC6A20* transcripts should be increased in PBMCs and/or extracellular-vesicle cargo; assess by targeted RT-qPCR or capture RNA-seq; (iii) Protein—where assayable, explore targeted immunoassays or MS-based panels; (iv) Performance—benchmark effect sizes by standardized z-scores, ROC/AUC, and longitudinal change versus clinical scales (e.g., UHDRS), with covariate adjustment (age, sex, CAG, PMI/hemolysis indices); (v) Validation—discovery/validation split, pre-registered analysis plan, and independent cohort replication. These steps convert computational convergence into testable biomarker hypotheses without over-extending the present dataset.

For clinical translation, *MIR219A2* quantification in CSF/plasma (small-RNA-seq or RT-qPCR) and monitoring of its up-regulated targets in PBMCs and/or extracellular-vesicle cargo are compatible with current HD biofluid biomarker frameworks [74,75]. In practice, these readouts would be evaluated alongside established trial-relevant endpoints—CSF/plasma neurofilament light (NfL) as a neurodegeneration marker and CSF mutant huntingtin (mHTT) as a disease-specific target-engagement marker [76,77,78,79]. Exploratory astroglial/inflammatory markers such as YKL-40 (CHI3L1) also show CSF elevations in HD and offer an orthogonal axis for triangulation [5,80]. Feasibility for small-RNA readouts in accessible matrices is supported by CSF microRNA studies in HD and by plasma EV small-RNA signatures in premanifest disease [81,82]. Accordingly, we envisage the *MIR219A2* axis as a complementary molecular layer to be integrated into multi-analyte panels (with standardized QC and longitudinal endpoints), rather than a replacement for existing biomarkers.

While this re-analysis is strictly in silico, we also specify concrete functional assays to test direct *MIR219A2*–target binding. First, dual-luciferase 3′UTR reporters for *FOXC1*, *NFKBIA*, *SLC38A2*, and *SLC6A20* should be assayed with *MIR219A2* mimic/inhibitor in human iPSC-derived cortical neurons and astrocytes, using wild-type versus seed-mutant reporters to demonstrate sequence-specific repression. Second, AGO2-CLIP/eCLIP (or AGO-RIP) in the same cells should detect *MIR219A2* engagement on these 3′UTRs (peaks/chimeras), with concordant qPCR/Western changes upon *MIR219A2* modulation. Appropriate controls include non-targeting miRNA, scrambled mimic, and a related miR-219 family member. A priori power (e.g., ≥20–30% effect at α = 0.05), biological replicates (n ≥ 3), and BH-FDR across multi-gene comparisons are recommended to ensure robust inference.

To avoid implying genome-wide regulator or pathway inference, we did not perform TF- or miRNA-regulator analyses across the entire BA9 dataset. Instead, our workflow uses two complementary in silico components: (i) a descriptive view of the compact core to illustrate directionality at the gene level; and (ii) a panel-independent, full-universe enrichment that tests validated *MIR219A2* target sets against all FDR-significant up-regulated BA9 genes (Results 2.6; Table 4). Beyond this, we present GO-BP bubbles only for visualization under relaxed thresholds, and we explicitly note that no term met FDR < 0.05 in the primary analysis; thus, no whole-transcriptome pathway claim is made. Consequently, any putative regulatory relationships are associative and hypothesis-generating pending experimental confirmation. (retain original citations)

### Limitations

Single-cell and spatial data are required to resolve cell-type assignments for *MIR219A2* and its targets because bulk BA9 profiles combine composition shifts and cell-intrinsic regulation with variance introduced by post-mortem factors [36]. The validated-target overlap was intentionally stringent and small; extending the intersection to the entire padj < 0.05 BA9 Up universe should increase counts and recover FDR-significant pathways/modules suitable for figure-grade networks. Finally, direct validation (AGO-CLIP, reporter assays) is warranted for *MIR219A2*–target pairs in human BA9, alongside testing regulator consistency (e.g., NF-κB) and solute-transporter modules in independent cohorts/regions.

Experimental validation and translational next steps. While this re-analysis is intentionally in silico, the present results specify falsifiable predictions:(1) *MIR219A2* should be reduced in BA9 (and potentially in CSF/plasma), concomitant with increased expression of FOXC1, NFKBIA, SLC38A2, and SLC6A20; (2) luciferase reporters bearing the native 3′UTRs of these targets should show *MIR219A2*-dependent repression that is abrogated by seed mutation; (3) AGO-CLIP (or AGO-RIP) in cortical or iPSC-derived systems should detect *MIR219A2* engagement on these transcripts; (4) single-nucleus or spatial assays should determine cellular provenance (glial/vascular vs. neuronal) of the *MIR219A2* axis. These constitute the prespecified next steps to convert computational convergence into mechanistic validation and assess biomarker potential.

## 4. Materials and Methods

### 4.1. Study Design and Dataset

We conducted a secondary analysis of the public RNA-seq dataset GSE64810 (NCBI Gene Expression Omnibus, Bethesda, MD, USA), which profiled post-mortem human BA9 (prefrontal cortex) from individuals with HD (*n* = 20) and neuropathologically normal controls (*n* = 49). In the originating study, BA9 tissue was obtained from the Harvard Brain and Tissue Resource Center, McLean Hospital (Belmont, MA, USA), the Human Brain and Spinal Fluid Resource Center, VA West Los Angeles Healthcare Center (Los Angeles, CA, USA), and the Banner Sun Health Research Institute (Sun City, AZ, USA); libraries were prepared with the TruSeq RNA Sample Prep Kit (Illumina, San Diego, CA, USA), RNA integrity was assessed on a 2100 Bioanalyzer (Agilent Technologies, Santa Clara, CA, USA), and pooled libraries were sequenced (2 × 101 bp) on a HiSeq 2000 at the Tufts University Core Facility (Boston, MA, USA); demultiplexing used the CASAVA pipeline (Illumina, San Diego, CA, USA). The original study reported that 19% (5480/28,087) of confidently detected genes were differentially expressed at FDR < 0.05 and were predominantly up regulated, with enrichments in immune response, neuroinflammation, and developmental (homeobox/HOX) programs, consistent with a multicellular response in BA9. (For replication in the originating report, RT-qPCR used the iScript cDNA Synthesis Kit (Bio-Rad Laboratories, Hercules, CA, USA) and TaqMan Gene Expression Assays (Life Technologies, Carlsbad, CA, USA) on an ABI 7900HT Real-Time PCR System (Applied Biosystems, Foster City, CA, USA).) We used these characteristics as contextual priors when interpreting our results. The biological plausibility of a miR-219 axis is strong: miR-219 is necessary and sufficient for oligodendrocyte differentiation and myelination, implying that its reduction could impair maturation/myelin maintenance in the cortex—a process increasingly linked to vulnerability to HD.

### 4.2. Preprocessing, Filtering, and Differential Expression

Gene-level expression and summary statistics were taken from the series-level resources. Before modeling, lowly expressed genes were filtered to reduce the multiple-testing burden. Group assignments (HD vs. control) were defined, and the primary differential-expression analysis was performed in GEO2R (NCBI Gene Expression Omnibus, Bethesda, MD, USA; https://www.ncbi.nlm.nih.gov/geo/geo2r; accessed 16 August 2025) using series-matrix inputs and the default limma-based linear-model framework with empirical-Bayes variance moderation and Benjamini–Hochberg false-discovery-rate adjustment; results were downloaded as tab-delimited tables for downstream ranking and visualization in R (R Foundation for Statistical Computing, Vienna, Austria). Unless otherwise specified, significance was defined as padj < 0.05.

### 4.3. Ranking and Visualization

To jointly capture effect size and statistical confidence, we prioritized genes by a composite ranking score S, defined as S = |log2FC| × (−log10(padj)), rather than imposing additional post hoc thresholds. Statistical significance was evaluated independently (padj < 0.05), and S was used strictly to order the significant genes and select candidates for display. For global visualization, we generated a volcano plot with points colored by significance and direction—significant up-regulated (red), significant down-regulated (blue), and non-significant (light gray). A horizontal reference line marks padj = 0.05 (i.e., −log10(padj) = 1.301), and the vertical reference at log2FC = 0 denotes no change. To anchor interpretation while avoiding clutter, we labeled the top 15 up- and top 15 down-regulated genes by S, using adaptive label offsets and reduced font size to minimize overlap. The composite score S was used only for prioritization/labeling and carries no standalone statistical meaning; all significance claims are based on BH-adjusted *p*-values. Volcano plots were generated in R (R Foundation for Statistical Computing, Vienna, Austria) using standard plotting utilities, and exported as vector graphics (PDF/SVG) for downstream inclusion.

### 4.4. Construction of the 80-Gene Core Set

For downstream analyses we defined a compact, direction-aware “core” signature. Among genes with padj < 0.05 genes, candidates were prioritized—not further thresholded—by S (as defined in Section 4.3), and the top 80 were retained while preserving up/down directionality to enable direction-aware enrichment and network tests. The target size (80) was pre-specified to balance pathway/PPI power and figure readability, and to avoid the overly generic enrichments that very large lists can yield. Gene symbols were harmonized to HGNC resources (HUGO Gene Nomenclature Committee, EMBL-EBI, Hinxton, Cambridgeshire, UK); duplicated or ambiguously mapped IDs were removed. Ties in the ranking were resolved first by smaller padj, then by larger |log2FC|. All steps were scripted in R (R Foundation for Statistical Computing, Vienna, Austria). No additional filters beyond padj < 0.05 were applied, and S was used strictly for ordering.

### 4.5. Validated Target Sets and Enrichment Testing

We compiled two independent MIR219A2 target resources: (i) miRTarBase (Chinese University of Hong Kong, Shenzhen, Guangdong, China; functional-only (luciferase reporter, qPCR, or Western blot ≥ 1; human symbols), and (ii) ENCORI/starBase CLIP-supported (Sun Yat-sen University, Guangzhou, Guangdong, China) (AGO-CLIP ≥ 1; hg38; human gene symbols).CLIP-supported targets for MIR219A2 (ENCORI/starBase) were intersected with the BA9 up-regulated universe (background = BA9 expressed genes), and enrichment was assessed by one-sided Fisher’s exact with Benjamini–Hochberg FDR. We report odds ratios (OR), 95% CIs (Haldane–Anscombe when needed), nominal p, and q values; full outputs (contingency table and overlap genes) are provided in Supplementary Table S11a.Targets were standardized to HGNC (EMBL-EBI, Wellcome Genome Campus, Hinxton, Cambridgeshire, UK), restricted to genes expressed in BA9 (background M), and intersected with the FDR-significant up-regulated universe (K). Enrichment used one-sided Fisher’s exact (greater); we report OR, 95% CI, *p*-values, and applied BH-FDR across the three pre-specified tests (functional-only, CLIP, validated-any). All analyses were executed in R (R Foundation for Statistical Computing, Vienna, Austria).

### 4.6. miRNA-Centered Integration (MIR219A2)

Motivated by the global upward shift of the BA9 signature, we pursued a single-miRNA–centered analysis focusing on MIR219A2, which we found significantly down-regulated in BA9. We curated experimentally validated targets for the mature forms hsa-miR-219a-5p, hsa-miR-219a-1-3p, and hsa-miR-219a-2-3p from DIANA-TarBase v9 (DIANA-Lab, University of Thessaly, Lamia, Greece; (https://dianalab.e-ce.uth.gr/tarbasev9; accessed 16 August 2025), using user-supplied CSV/XLSX exports. Gene symbols were harmonized to HGNC (EMBL-EBI, Wellcome Genome Campus, Hinxton, Cambridgeshire, UK) format, duplicates were collapsed, and targets were restricted to human gene symbols. We then intersected the consolidated target set with the up-regulated portion of the BA9 signature—first with the 80-gene core, and, if additional power was required, with the complete padj < 0.05 up-regulated universe. All steps were scripted in R (R Foundation for Statistical Computing, Vienna, Austria).

### 4.7. Transcription Factor Co-Regulation Analyses

Transcription factor (TF) co-regulation was assessed using two orthogonal resources. (i) TRRUST v2 (human) was queried on the MIR219A2 CLIP-overlap subset (*n* = 138 genes) to list TFs with multiple targets in the subset and to provide target-level transparency (Supplementary Table S12). TRRUST v2 is maintained by groups at Yonsei University, Seoul, Republic of Korea, and Korea University, Seoul, Republic of Korea. (ii) MSigDB C3:TFT (Gene Symbols) from the Broad Institute of MIT and Harvard, Cambridge, MA, USA was used for formal over-representation testing against the BA9 expressed-gene background (universe = genes with non-missing padj; “Up” = padj < 0.05 & log2FC > 0). Enrichment was evaluated by one-sided Fisher’s exact (greater) with Benjamini–Hochberg FDR control within the TFT collection; we report OR, 95% CI (Haldane–Anscombe when needed), p, and q (Supplementary Table S11b). All findings are associative (non-causal). Analyses were executed in R (R Foundation for Statistical Computing, Vienna, Austria).

### 4.8. Cell-Type Marker Context and Sensitivity Analyses

Cell-type marker panels for astrocytes, microglia, oligodendrocytes, endothelial cells, and neurons were compiled from curated brain marker resources, including PanglaoDB (Integrated Cardio Metabolic Centre, Karolinska Institutet, Huddinge, Sweden), CellMarker 2.0 (College of Bioinformatics Science and Technology, Harbin Medical University, Harbin, Heilongjiang, China), and NeuroExpresso (Pavlidis Lab, University of British Columbia, Vancouver, BC, Canada). FDR-significant BA9 sets were intersected with each panel to provide a descriptive cellular context. For sensitivity analyses, MIR219A2 enrichment was re-evaluated using (i) a background excluding the union of all marker genes and (ii) stratified backgrounds (neuron-enriched vs. glia-enriched). Enrichment testing used one-sided Fisher’s exact with Benjamini–Hochberg FDR control, as in the main analysis; computations were performed in R (R Foundation for Statistical Computing, Vienna, Austria).

### 4.9. Computational Validation and Robustness Criteria

Computational validation criteria were defined a priori as the convergence of three elements: (i) orthogonal evidence for the same miRNA–mRNA relationship (miRTarBase (School of Medicine, The Chinese University of Hong Kong, Shenzhen, Guangdong, China) functional assays and ENCORI/starBase (Sun Yat-sen University, Guangzhou, Guangdong, China) AGO-CLIP), (ii) direction-aware enrichment consistent with the BA9 contrast (down-regulated miRNA versus up-regulated mRNAs), and (iii) robustness to composition confounding, assessed by repeating tests after excluding the union of canonical CNS markers and within neuron- versus glia-enriched backgrounds (Supplementary Tables S8 and S9). No wet-lab assays were performed in this study. Cell-type context was addressed by annotating FDR-significant sets with canonical CNS marker panels (astrocyte, microglia, oligodendrocyte, endothelial, neuron) and by re-testing enrichment in marker-excluded and stratified (neuron/vs. glia) backgrounds. No deconvolution was performed; analyses remain conservative by design. No joint TF–miRNA causal modeling or genome-wide TF/miRNA regulator inference (e.g., motif/regulon modeling) was undertaken; all inferences are associative rather than causal. Regulator claims are limited to validated MIR219A2 target-set enrichment against the full BA9 FDR up-regulated background (Results 2.6; Table 4).

### 4.10. Functional Enrichment, PPI, and Regulator Analysis

For the overlap between validated *MIR219A2* targets and BA9 up-regulated genes, we performed over-representation analysis (ORA) with false discovery rate (FDR) control against Gene Ontology (GO) (Gene Ontology Consortium; accessed via geneontology.org and QuickGO at EMBL-EBI, Hinxton, UK; all accessed 16 Aug 2025), KEGG (Bioinformatics Center, Institute for Chemical Research, Kyoto University, Uji, Kyoto, Japan; accessed 16 Aug 2025), and Reactome (Ontario Institute for Cancer Research, Toronto, Canada; EMBL-EBI, Hinxton, UK; Cold Spring Harbor Laboratory, Cold Spring Harbor, NY, USA; all accessed 16 Aug 2025), and queried STRING (minimum required interaction score of 0.7) to evaluate protein–protein interactions, followed by a preliminary module/hub inspection. STRING is developed by the STRING Consortium including SIB Swiss Institute of Bioinformatics, Lausanne, Switzerland; University of Zurich, Zurich, Switzerland; EMBL, Heidelberg, Germany; and Novo Nordisk Foundation Center for Protein Research, University of Copenhagen, Copenhagen, Denmark. Bubble plots were produced in R (R Foundation for Statistical Computing, Vienna, Austria) with relaxed display thresholds to summarize trends; inferential claims relied on FDR < 0.05, under which no GO-BP term was significant. Because the overlap comprised only four genes, the ORA and PPI outputs were treated as exploratory and are reported qualitatively, acknowledging limited statistical power. Applying the same workflow to the full 80-gene core may increase power and yield more interpretable, FDR-controlled enrichments and network structure. 

Two distinct settings were used: (i) primary inferential ORA—BA9 expressed-gene background; one-sided Fisher; Benjamini–Hochberg FDR across GO/KEGG/Reactome—for formal calls; and (ii) STRING GO–Biological Process visualization—default STRING background and relaxed display thresholds—for trend summarization in Figure 4. Only the primary ORA was used for inference; see Supplementary Table S10 for the primary ORA parameters and outcome (no GO-BP term significant at FDR < 0.05). This subsection is entirely in silico; no chemicals, reagents, devices, instruments, or commercial cell lines/samples/materials were used, so manufacturer city/state/country disclosures are not applicable beyond the database/software attributions above. 

## 5. Conclusions

Re-analysis of BA9 confirmed the BH–FDR-significant counts within the 80-gene core (74 up, 6 down); transcriptome-wide, the BA9 contrast shows a modest predominance of up-regulation (Figure 1). Within the down-regulated subset, *MIR219A2* showed strong depletion, and intersecting its experimentally validated targets with the four core identified up-regulated BA9 genes—*FOXC1*, *NFKBIA*, *SLC38A2*, *SLC6A20*—consistent with target derepression when *MIR219A2* is reduced. As expected for a four-gene overlap, ORA across GO/KEGG/Reactome did not reach FDR significance, and STRING returned no edges, indicating the absence of a cohesive high-confidence interaction module at this stringency. Taken together, these analyses support a BA9-specific, MIR219A2-aligned association signal spanning inflammatory, neurovascular/glial, and transport/synaptic facets; we regard this signal as hypothesis-generating pending experimental confirmation (Section 2.6; Table 4).

Major limitations include the mixing of cell types inherent to bulk tissue profiling, the regional specificity of the analysis (BA9), and the small size of the *MIR219A2* target overlap within the 80-gene core. Priorities for future work are to increase statistical power by testing *MIR219A2* targets against the entire BA9 up-regulated universe (padj < 0.05), to localize effects with single-cell and spatial transcriptomic approaches, and to establish causality through experimental validation, including AGO-CLIP and reporter assays that directly confirm *MIR219A2*–target interactions.

## Figures and Tables

**Table 1:** Top 15 up-regulated genes in BA9 (ranked by S).

Symbol	log2FC	padj	S
*SLC16A12*	4.094	5.670 × 10^−15^	58.320
*SIX1*	3.689	2.400 × 10^−16^	57.615
*OGN*	4.372	2.850 × 10^−13^	54.842
*SLC26A7*	3.646	9.690 × 10^−16^	54.747
*BMP5*	4.593	1.500 × 10^−12^	54.305
*SPTLC3*	3.086	5.670 × 10^−15^	43.968
*CLIC6*	3.667	2.830 × 10^−12^	42.343
*WDR86-AS1*	3.294	2.850 × 10^−13^	41.327
*VNN2*	2.913	1.880 × 10^−14^	39.985
*DSP*	2.976	4.300 × 10^−14^	39.779
*SLC6A20*	2.710	3.750 × 10^−15^	39.088
*CRABP1*	2.648	3.750 × 10^−15^	38.201
*SIX2*	3.994	4.910 × 10^−10^	37.182
*PTGDR*	3.051	4.460 × 10^−12^	34.628
*OMD*	2.385	6.030 × 10^−15^	33.911

**Table 2:** Top 15 down-regulated genes in BA9 (ranked by S).

Symbol	log2FC	padj	S
*MIR219A2*	−3.005	2.080 × 10^−8^	23.088
*TMEM88B*	−2.764	5.730 × 10^−9^	22.781
*SAXO1*	−2.092	4.760 × 10^−9^	17.414
*TNIP3*	−2.343	1.220 × 10^−6^	13.856
*SYT2*	−1.870	6.900 × 10^−8^	13.392
*MTRNR2L8*	−2.514	8.220 × 10^−6^	12.784
*SLCO5A1*	−1.955	9.100 × 10^−7^	11.813
*GPD1*	−1.293	2.040 × 10^−9^	11.236
*IL17REL*	−2.056	6.570 × 10^−6^	10.655
*CARNS1*	−1.920	3.330 × 10^−6^	10.515
*PLAC9P1*	−2.069	9.860 × 10^−6^	10.360
*RAET1E-AS1*	−1.436	2.390 × 10^−7^	9.511
*LINC01310*	−1.412	2.720 × 10^−7^	9.269
*C2CD4D*	−1.148	1.240 × 10^−8^	9.078
*KLC2-AS2*	−1.487	7.920 × 10^−7^	9.074

log2FC: log2-transformed fold change (HD vs. control; positive = up in HD, negative = down). padj (BH): Benjamini–Hochberg false-discovery-rate–adjusted *p*-value (significance threshold padj < 0.05 unless stated otherwise). S: composite ranking score used for prioritization, S = ∣log2(FC)∣×(−log10(padj)); a larger S indicates a larger effect size and stronger statistical support; however, S is not a stand-alone significance test.

**Table 3:** *MIR219A2* validated targets overlapping BA9 up-regulated genes (GSE64810).

Symbol	log2FC	padj (BH)
*FOXC1*	1.762	4.06 × 10^−9^
*NFKBIA*	1.127	1.43 × 10^−12^
*SLC38A2*	1.257	4.02 × 10^−12^
*SLC6A20*	2.710	3.75 × 10^−15^

log2FC: log2-transformed fold change (HD vs. Control; positive = up in HD). padj (BH): Benjamini–Hochberg FDR-adjusted *p* value.

**Table 4:** Enrichment of *MIR219A2* target sets against the BA9 up-regulated universe (FDR < 0.05). Background = expressed BA9 genes; one-sided Fisher (greater); BH-FDR across three tests.

Target Set	Size (*n*)	Overlap (a)	OR	95% CI	p (One-Sided)	q (BH)
miRTarBase functional-only (luc/qPCR/Western)	8	4	6.57	[1.64, 26.27]	0.0137	0.0137
ENCORI/starBase CLIP-supported	640	138	1.84	[1.52, 2.23]	2.56 × 10^−9^	7.68 × 10^−9^
miRTarBase validated any (functional ∪ CLIP)	42	15	3.66	[1.94, 6.88]	1.87 × 10^−4^	1.87 × 10^−4^

Results were direction-consistent and significant (Table 4): miRTarBase functional-only, overlaps = 4, OR = 6.57, 95% CI [1.64, 26.27], p = 0.0137, q = 0.0137; ENCORI/StarBase CLIP-supported, overlaps = 138, OR = 1.84, 95% CI [1.52, 2.23], p = 2.56 × 10^−9^, q = 7.68 × 10^−9^; validated-any, overlaps = 15, OR = 3.66, 95% CI [1.94, 6.88], p = 1.87 × 10^−4^, q = 1.87 × 10^−4^.

## Data Availability

Data are contained within the article.

## Supplementary Materials

The following supporting information can be downloaded at: https://www.mdpi.com/article/10.3390/ijms26188934/s1.
